# Astragaloside-IV alleviates high glucose-induced ferroptosis in retinal pigment epithelial cells by disrupting the expression of miR-138-5p/Sirt1/Nrf2

**DOI:** 10.1080/21655979.2022.2049471

**Published:** 2022-03-18

**Authors:** Xuyuan Tang, Xiuyi Li, Dongyan Zhang, Wei Han

**Affiliations:** a Department of Ophthalmology, the First Affiliated Hospital, Zhejiang University School of Medicine, Hangzhou, Zhejiang, China; b Department of Ophthalmology, Eye Center of the Second Affiliated Hospital, School of Medicine, Zhejiang University, Hangzhou, China

**Keywords:** DR, diabetic retinopathy, RPE, retinal pigment epithelial, AS-IV, astragaloside-IV

## Abstract

Astragaloside-IV (AS-IV) (C_41_H_68_O_14_) is a high-purity natural product extracted from *Astragalus*, which has demonstrated biological activities. However, the effect of AS-IV on retinal pigment epithelial (RPE) cells in diabetic retinopathy (DR) remains unclear. In this study, high glucose (HG) was shown to promote ARPE-19 RPE cell death, increase the contents of reactive oxygen species (ROS) and oxidized glutathione (GSSG), and enhance lipid peroxidation density of mitochondrial membrane. In contrast, AS-IV decreased glutathione (GSH) content, mitochondria size and ridge. Addition of iron death inhibitor Ferrostatin-1 (Fer-1) to RPE cells decreased cell dead rate, thus indicating that HG-induced mitochondrial damage occurred due to ferroptosis. AS-IV alleviated HG-induced RPE cell damage. Furthermore, HG decreased levels of silent information regulator 1 (Sirt1) and nuclear factor (erythroid-derived 2)-like 2 (Nrf2) in the nucleus of RPE cells; AS-IV could alleviate these effects and increased expression of glutathione peroxidase 4 (GPX4), glutamate cysteine ligase (GCLM) and glutamate cysteine ligase catalytic subunit (GCLC), which are Nrf2 downstream genes. Mechanistically, AS-IV was shown to alleviate the effects of HG by increasing mir-138-5p expression in RPE cells and promoting expression of Sirt1 and Nrf2 in the nucleus. Transfection of mir-138-5p agonist inhibited the regulatory effects of AS-IV on Sirt1 and Nrf2, accompanied by decreased GPX4, GCLM and GCLC levels, and restoration of ferroptosis-related changes. Collectively, HG increased ferroptosis rate in RPE cells. In addition, AS-IV inhibited miR-138-5p expression, subsequently increasing Sirt1/Nrf2 activity and cellular antioxidant capacity to alleviate ferroptosis, resulting decreased cell death, which potentially inhibits the DR pathological process.

## Graphical abstract

**Figure:**
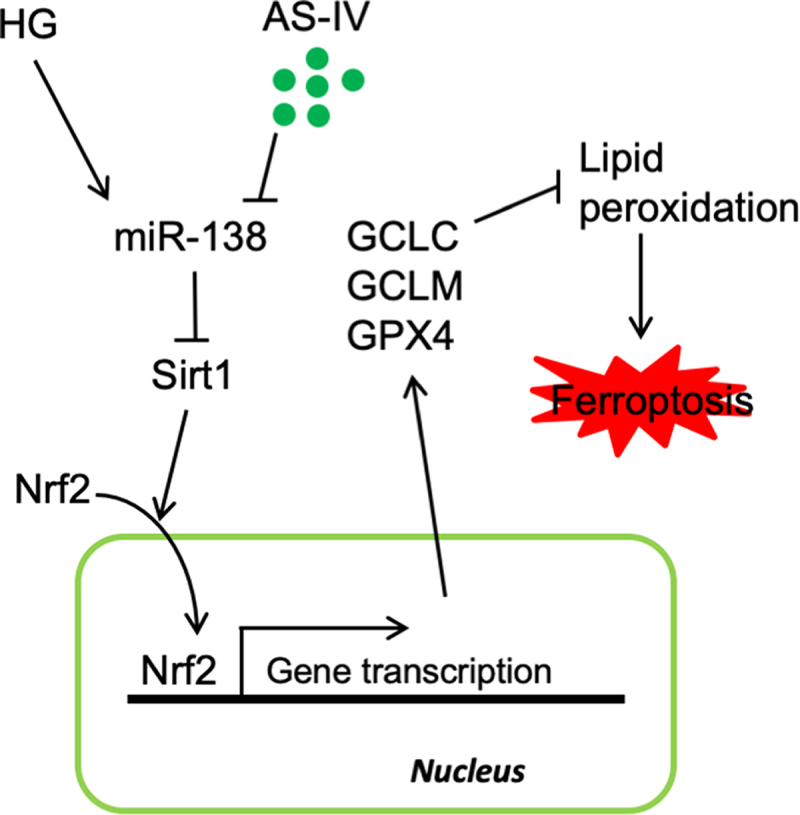

## Introduction

Diabetic retinopathy (DR) is an eye disorder manifested as a consequence of diabetic microangiopathy and neuropathy, being considered the main cause of blindness in adults. The proportion of DR in diabetic patients is 24.7–35.7%. Current treatments for DR, including anti-angiogenic drug administration, laser therapy, and surgical therapy, are often unsatisfactory, and new treatments are urgently needed.

Retinal segment endothelial (RPE) cells are located between photoreceptor cells and choroidal capillary layer in the eye, thus forming the blood retina barrier (BRB) outside the retina [1,2]. These cells are involved in the uptake of ions, water and nutrients, as well as the secretion of metabolic waste, therefore being crucial for retinal function maintenance. One of the main alterations related to DR is the destruction of BRB as a consequence of damage to RPE cells [2,3]. Therefore, understanding the mechanisms underlying RPE cell damage is paramount to elucidate the pathological mechanism of DR in order to develop new therapeutic strategies.

The activity of RPE cells is conditioned to satisfactory energy supply thus requiring high metabolic activity and mitochondrial content in cells. The production of reactive oxygen species (ROS) follows mitochondrial energy production, hence ROS production level in RPE cells is high [4,5]. Under normal conditions, the antioxidant system in cells can remove excessive ROS. However, ROS production level increases in DR because the activity of the antioxidant system decreases, with hyperglycemia as the main cause leading to this phenomenon, being also one of the causes for the loss of vitality and function of RPE cells [6,7].

Ferroptosis is characterized by iron-dependent cell death resultant from increased lipid peroxidation. It has been reported that RPE cell death occurs in age-related macular degeneration. Interestingly, the addition of ferroptosis inhibitor Ferrostatin-1 (Fer-1) and iron chelator deferoxamine has been shown to effectively inhibit events triggering cell death induced by decreased levels of glutathione (GSH) or treatment with t-BH, being therefore more effective than treatment with necrosis and apoptosis inhibitors [8,9]. In patients with DR, ROS and GSH levels are increased, while the levels of superoxide dismutase (SOD) are decreased. However, whether ferroptosis occurs in RPE cells during DR and how it might influence cell death remain largely unexplored.

**Figure 1.**
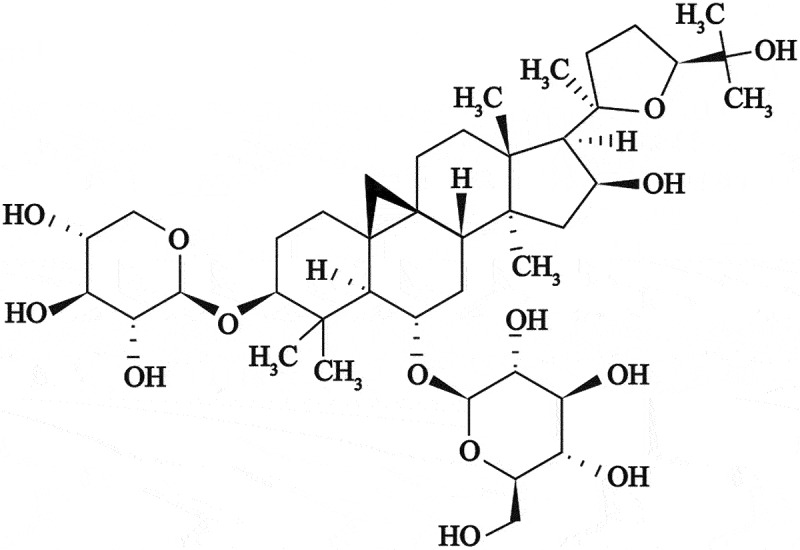
Chemical structure of astragaloside-IV (AS-IV) obtained from *Astragalus membranaceus.*

Astragaloside-IV (AS-IV) (C_41_H_68_O_14_) is a high-purity natural product extracted from *Astragalus membranaceus* (Figure 1) with multiple demonstrated biological activities, including immune system boosting, anti-apoptotic, anti-stress and anti-oxidative [^10–13^]. Previous studies showed that AS-IV can inhibit death of retinal ganglion and endothelial cells as well as restore cell function, and whose underlying mechanism involved the anti-oxidant effect of AS-IV. Thus, AS-IV seems to effectively mitigate the pathological process leading to DR [14,15]. Importantly, AS-IV has been shown to effectively inhibit death of RPE cells during streptozocin-induced DR; changes occurring at cellular level showed that AS-IV can inhibit cell damage induced by high glucose levels by increasing the expression of miR-128 [16]. Therefore, it would of interest to determine whether AS-IV can mitigate damage to RPE cells by inhibiting ferroptosis.

Nuclear factor (erythroid-derived 2)-like 2 (Nrf2) is a member of the Cap’n’Collar subfamily of basic leucine zipper transcription factors. Under oxidative stress, Nrf2 enters the nucleus, binds to antioxidant response elements (ARE) found in the promoter region of several related genes, thereby promoting the expression of target genes such as glutathione peroxidase (*GPX*), hemeoxygenase 1 (*HO-1*), glutamate cysteine ligase catalytic subunit (*GCLC*) and *GCM*, thus playing a key role in maintaining the antioxidative/oxidative balance in the cells [17]. In DR, Nrf2 levels are downregulated in RPE cells, and activation of Nrf2 can effectively inhibit damage induced by high glucose on RPE cells [18,19]. Importantly, Nrf2 has been shown to inhibit ferroptosis in many diseases by promoting expression of antioxidant enzymes. However, it has not been reported whether AS-IV can regulate the level of expression and activity of Nrf2, thus affecting the function of RPE cells. However, in HK-2 cells and in a model of myocardial ischemia/reperfusion injury, AS-IV inhibited cell injury by increasing the level of expression and activity of Nrf2 [20,21].

Silent information regulator 1 (Sirt1), a nicotinamide adenine nucleotide (NAD)-dependent protein deacetylase, has been shown to positively regulate the level of expression and activity of Nrf2 and participate in the process of cisplatin-induced nephrotoxicity, myocardial ischemia/reperfusion, and tumor drug resistance [22–24]. AS-IV has been shown to increase the level of Sirt1 in cells and participate in the inhibition of high glucose-induced mesangial cell activation, podocyte epithelial-mesenchymal transformation, and cardiomyocyte injury [25–27]. In high glucose- or H_2_O_2_-treated RPE cells, the level of Sirt1 decreases [28]. Considering the previous findings, it can be speculated that AS-IV might increase the level of expression and activity of Nrf2 in RPE cells by increasing the level of Sirt1. Moreover, it is known that the level of Sirt1 is regulated by miRNA expression in RPE cells [29,30], and changes in miRNA abundance are one of the key factors of ‘metabolic memory’ in diabetes-related complications, including RPE cell injury in DR [31,32]. Moreover, AS-IV can affect a variety of biological processes by regulating miRNA expression levels, including that of miR-34a and Sirt1, thereby protecting cardiomyocytes [25]. Thus, investigating whether AS-IV can affect the level and activity of Sirt1/Nrf2 by regulating miRNA expression levels might help to elucidate the pathological process of DR.

Thus, it can be hypothesized that AS-IV might relieve RPE cell damage via the Sirt1/Nrf2 signaling cascade mediated by ferroptosis. Therefore, the aims of this study were: i) to investigate changes related to ferroptosis in high glucose-exposed RPE cells; ii) to demonstrate whether AS-IV can regulate the function of RPE cells by inhibiting ferroptosis; iii) to explore changes in the expression of miRNAs in cells under high glucose; iv) to explore whether AS-IV can increase Sirt1 level and enhance level of expression and activity of Nrf2 by regulating expression of miRNAs; v) to demonstrate whether AS-IV can inhibit ferroptosis by miRNA/Sirt1/Nrf2 signaling cascade thereby alleviating the damage induced by high glucose in RPE cells.

## Materials and methods

### Cells and reagents

ARPE-19 cells were purchased from Procell Life Science & Technology Co. Ltd., China (#CL-0026; preservation institution: ATCC, CRL-2302, BCRC), cultured in 90% dulbecco’s modified eagle medium (DMEM, #PM150210; Procell) with 10% FBS (#164210-500; Procell) at 37°C and 5% CO_2_. Fer-1 (#T6500; Target Molecule Corp., Boston, MA, USA), erastin (#T1765; TargetMol), AS-IV (#HY-N0431; MCE, USA), and EX 527 (#T6111; TargetMol) were used in the experiments.

### Cell viability assay

High glucose was represented by treatment with 25 mM glucose for 48 h, and mannitol (25 mM) was used as the solvent (glucose final concentration in the medium was 5.5 mM). Cells were digested with 0.25% trypsin dissolved in EDTA and collected; cells were then washed twice with PBS and submitted to centrifugation at 1,200 rpm for 5 min. According to manufacturer’s instructions for the calcein-AM/propidium iodide (PI) cell death detection kit (Solarbio, China, CA1630), 500 μL of binding buffer was added to resuspend cells, and 5 μL of calcein-AM/PI solution was added to the mixture followed by proper homogenization, and the reaction was carried out for 15 min at room temperature (with negative control). A flow cytometer (CytoFLEX; Beckman Coulter, USA) was used for determining cell viability based on fluorescence. Calcein-positive cells (calcein+) indicated living cells and PI-positive cells (PI+) indicated dead cells. Percentage of dead cells was calculated according to the formula: calcein^−^PI^+^/(calcein^+^PI^−^+calcein^−^PI^+^)×100%.

### Levels of lipid oxidation and ROS

Cells were digested with 0.25% trypsin in EDTA, collected, and centrifuged at 1,500 rpm for 5 min, and the supernatant was removed. Cells were washed twice with PBS and centrifuged at 1,500 rpm for 5 min. Diluted BODIPYTM 581/591 C11 lipid oxidation probe (#D3861; Invitrogen, USA) or CM-H2DCFDA fluorescent probe (#D6470; Solarbio, China) was added to the cells according to the manufacturer’s instructions. Cells were incubated in 37°C for 20 min, mixed every 3 min, and washed thrice with serum-free medium. Then, 500 μL of PBS was used to resuspend the cells, and the levels of lipid oxidation and ROS were determined by in a CytoFLEX flow cytometer (Beckman Coulter).

### GSH and GSSG levels

According to the manufacturer’s instructions for GSH and GSSG assay kit (#S0053; Beyotime, China), the working solution and samples were prepared. Sample and standard solutions were then thoroughly mixed, and 150 μL of total glutathione test solution was added to the mixture followed by homogenization and incubation at room temperature for 5 min. Subsequently, 50 μL of 0.5 mg/mL NADPH solution was added to the mixture followed by homogenization, and the mixture was left to react for 25 min, after which it was put in a microplate reader to determine total glutathione content by measuring sample absorbance at 412 nm. GSSG content was determined after removing GSH from samples with appropriate reagents. GSH content was calculated by deducting GSSG content from total glutathione content (GSSG and GSH).

### Transmission electron microscopy (TEM) analysis

Cells were fixed with 2.5% glutaraldehyde for 3 h, scraped, and collected. After centrifugation at 1,000 rpm, the cells were fixed with fresh 2.5% glutaraldehyde (#A17876; Alfa Aesar, USA) at 4°C for 3 h. Subsequently, cells were washed with 0.1 M phosphate buffer (pH 7.4) three times, 15 min each time. Cells were fixed with 1% osmic acid 0.1 M phosphate buffer (pH 7.4) for 2 h at room temperature, and washed with 1 M phosphate buffer (pH 7.4) for three times, 15 min each time. Cells were then dehydrated using a gradient concentration of ethanol solution (50, 70, 80, 90, 95, 100%), 15 min with each solution. The mixture was submitted to treatment with penetrating solution (acetone:Epon 812 mixture, 1:1, *v/v*) overnight. Then, the mixture was polymerized at 60°C for 48 h, and then cut into 70-nm thin sections. Uranium-lead double staining (2% uranium acetate saturated aqueous solution, lead citrate, for 15 min) was performed to dehydrate slices overnight at room temperature, and samples were observed in a Tecnai G20 TWIN transmission electron microscope (FEI Company, USA).

### Nuclear protein isolation

CelLytic™ NuCLEAR extraction kit (#P0027; Beyotime) was used for nuclear protein isolation. Cell culture medium was removed, and pre-cooled PBS was used to wash cells. Cells were transferred to EP tubes, submitted to centrifugation, and the supernatant was discarded. Subsequently, 200 μL of cytoplasmic protein extraction reagent A (with PMSF) was added to 20 μL of cell pellet, resuspended and dispersed by vortexing for 5 s at maximum speed, and placed in an ice bath for 15 min. Then, 10 μL of cytoplasmic protein extraction reagent B was added to the mixture, followed by vortexing at maximum speed for 5 s, placed in an ice bath for 1 min, followed by vortexing at maximum speed for 5 s and centrifugation at 4°C at 15,000 g for 5 min, and the resulting supernatant was the extracted cytoplasmic protein, being transferred to a pre-cooled plastic tube. For protein precipitation, 50 μL of nuclear protein extraction reagent (with PMSF) was added to the cell pellets, and the mixture was vortexed at maximum speed for 30s, resuspended, and the mixture was placed in an ice bath. Subsequently, the mixture was vortexed at maximum speed for 30s every 2 min to a total time of 30 min. Then, the mixture was centrifugated at 4°C at 15,000 × *g* for 10 min. The resulting supernatant was immediately transferred to a pre-cooled plastic tube, being considered the extracted nuclear protein.

### Western blot analysis

Treated cells were washed with PBS, and lysis buffer (#P0013b; Beyotime) containing PMSF (#329-98-6; Nanjing Wohong, China) was added to the cells, and the mixture was left to react on ice for 30 min. After lysis, cell fragments and lysate were transferred to a 1.5-mL centrifuge tube and submitted to centrifugation at 12,000 rpm for 15 min at 4 °C. Protein concentration was measured by the Bradford method (#5000006; Bio-Rad Laboratories, USA). After protein concentration was determined, 30 μg of protein was mixed with 5 μL of loading buffer (15 g of SDS, 15.6 mL of 2M Tris pH 6.8, 57.5 g of glycerol, 16.6 mL of β-mercaptoethanol), followed by heat treatment for 8 min to enable denaturation, then cooled and centrifuged. Samples were loaded in 10% SDS-PAGE gel, separated by electrophoresis and transferred to a PVDF membrane (#162-0177; Bio-Rad). After sealing, 4% skim milk containing 0.1% Tween-20 was added onto the membrane, and primary antibodies (Sirt1, 1:1000, #ab189494, Abcam, UK; Nrf2, 1:1000, #ab89443, Abcam; LaminB, 1:1000, #ab16048, Abcam; GPX4, 1:1000, #ab51944, Abcam; GCLC, 1:2000, #ab207777, Abcam; GCLM, 1:1000, #ab126704, Abcam; GAPDH, 1:2500, #ab9485, Abcam) were added onto the membrane, followed by overnight incubation at 4 °C. The membrane was washed three times with PBS solution containing 0.1% Tween-20, and the HRP-labeled secondary antibody (Dianova, Germany) prepared with 4% skim milk containing 0.1% Tween-20 was added to the membrane, followed by incubation for 2 h at room temperature. Then, the membrane was washed three times with PBS solution containing 0.1% Tween-20, and the ECL developer (#170-5060; Bio-Rad) was added to the membrane. GelDoc Imaging System (Bio-Rad) was used for visualization of Western blots. Protein expression levels were normalized against GAPDH/LaminB.

### qRT-PCR analysis

RNA was extracted from cells using the RNeasy Minikit (#74106; QIAGEN, Germany) following the manufacturer’s instructions. Obtained RNA was treated with DNase with an RNase free DNase set (#79254; QIAGEN). A large-capacity cDNA reverse transcription kit (#4368813; Applied Biosystems, USA) was used for cDNA synthesis.

qRT-PCR was carried out with a SYBR® Green kit (Applied Biosystems) in a StepOnePlus™ Real-Time PCR System (Thermo Fisher Scientific, USA. C_T_ values were compared using the 2^−ΔΔC^_T_ method, and relative expression of miRNA in different samples was determined by using the U6 snRNA for normalization. qRT-PCR amplification conditions were: 95°C for 3 min; 95°C for 20 s, followed by 40 cycles of 55°C for 20 s. Primers used in reactions were miR-22-3p (5’-TGCGGCAAGCTGCCAGTTGAA-3’), miR-138-5p (5’-TGCGGCAGCTGGTGTTGTGAATC-3’), miR-199b-5p (5’-TGCGGCCCCAGTGTTTAGACTAT-3’), miR-211-5p (5’-TGCGGCTTCCCTTTGTCATCCT-3’), miR-212-3p (5’-TGCGGCTAACAGTCTCCAGTC-3’), miR-217-5p (5’-TGCGGCTACTGCATCAGGAACTG-3’), universal primer (5’-GTGCAGGGTCCGAGGT-3’), U6_F (5’-CTCGCTTCGGCAGCACA-3’), U6_R (5’-AACGCTTCACGAATTTGCGT-3’).

### Statistical analysis

Data are presented as mean ± standard deviation. Prism 9 (GraphPad Software Inc., USA) was used for data analysis. One-way analysis of variance (ANOVA) with least significant difference (LSD) was used for pairwise comparisons between group means. Student’s t test was used to determine differences between groups, and *P*-values < 0.05 were considered as statistically significant.

## Results

Astragaloside IV (AS-IV), a high purity natural product extracted from Astragalus, has the functions of maintaining immune balance, anti-stress, anti-oxidation and anti-apoptosis. The present study is aimed to explore its effects on retinal pigment epithelial cells (RPE) in diabetic retinopathy (DR). Meanwhile, we investigated the association between RPE cell damage and Sirt1/Nrf2 signaling cascade-regulated ferroptosis, and which miRNAs were involved in the process.

### HG promotes ferroptosis in RPE cells

**Figure 2.**
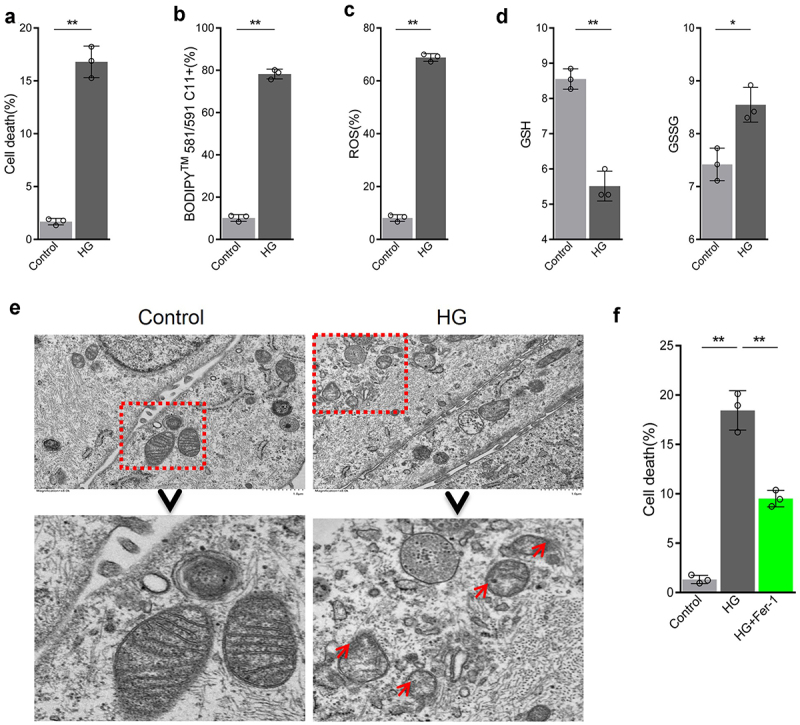
High glucose promotes ferroptosis in retinal pigment endothelial (RPE) cells. (a) Cell viability determined by calcein-AM/propidium iodide (PI) staining. (b) Intracellular lipid oxidation rate determined using the C11-BODIPY™ 581/591 probe. C) Total intracellular content of reactive oxygen species (ROS) detected by the CM-H2DCFDA probe. (d) Glutathione (GSH) and oxidized glutathione (GSSG) assay. (e) Cell substructure determined by transmission electron microscopy (TEM); scale bar: 1.0 μm. (f) Cell death rate analyzed by calcein-AM/PI staining. * indicates *p* values < 0.05; ** indicates *p* values < 0.01.

Firstly, ARPE-19 cells were treated with 25 mM glucose to simulate a high glucose condition found in DR. High glucose could increase death rate of ARPE-19 cells (Figure 2(a)). In addition, further experiments showed that high glucose increased levels of lipid peroxidation as well as ROS and GSSG production, whereas decreased GSH content (Figure 2(b–d)). In addition, mitochondria were reduced in size, the mitochondrial ridge decreased or were absent, the outer mitochondrial membrane was disrupted, and membrane density in certain areas was increased (Figure 2(e)). After the addition of ferroptosis inhibitor Fer-1 to cells exposed to high glucose conditions, cell death rate was decreased (Figure 2(f)). Collectively, these results indicate that high glucose can increase cell death rate of RPE cells by promoting ferroptosis.

### AS-IV inhibits ferroptosis induced by high glucose in RPE cells

**Figure 3.**
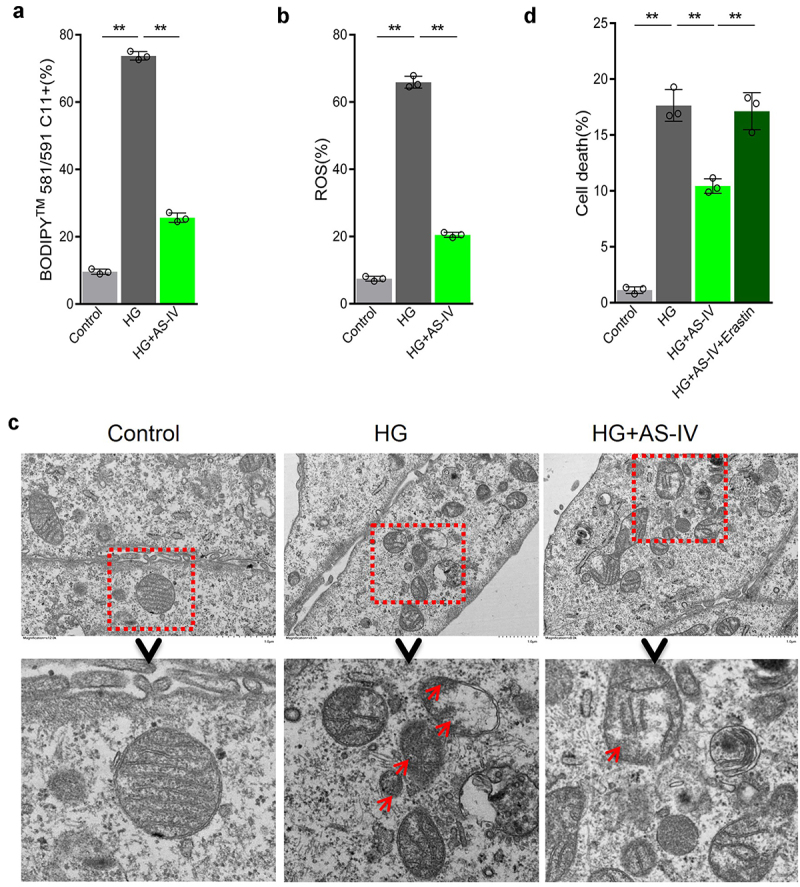
Astragaloside-IV (AS-IV) inhibits ferroptosis induced by high glucose in retinal pigment endothelial (RPE) cells. (a) Intracellular lipid oxidation level measured by the C11-C11-BODIPY™ 581/591 probe. (b) Total intracellular content of reactive oxygen species (ROS) detected with the CM-H2DCFDA probe. (c) The cell substructure observed by TEM; D. The level of cell death analyzed by calcein-AM/propidium iodide (PI) staining. * indicates *p* values < 0.05; ** indicates *p* values < 0.01.

The inhibiting effect of AS-IV on ferroptosis in RPE cells under high glucose conditions was evaluated. Compared with high glucose-treated group, levels of lipid peroxidation and ROS in AS-IV-treated cells decreased (Figure 3(a,b)), and morphological mitochondrial alterations were repaired (Figure 3(c)). These results indicated that AS-IV could inhibit ferroptosis in RPE cells exposed to high glucose conditions. Further experiments revealed that AS-IV decreased death rate of RPE cells under high glucose conditions, and the ferroptosis inducer erastin could antagonize this effect (Figure 3(d)), indicating that AS-IV could reduce cell death rate by inhibiting the negative effect of high glucose-induced ferroptosis in RPE cells.

### AS-IV inhibits the negative effect of high glucose to reduce Sirt1/Nrf2 signal activity in RPE cells

**Figure 4.**
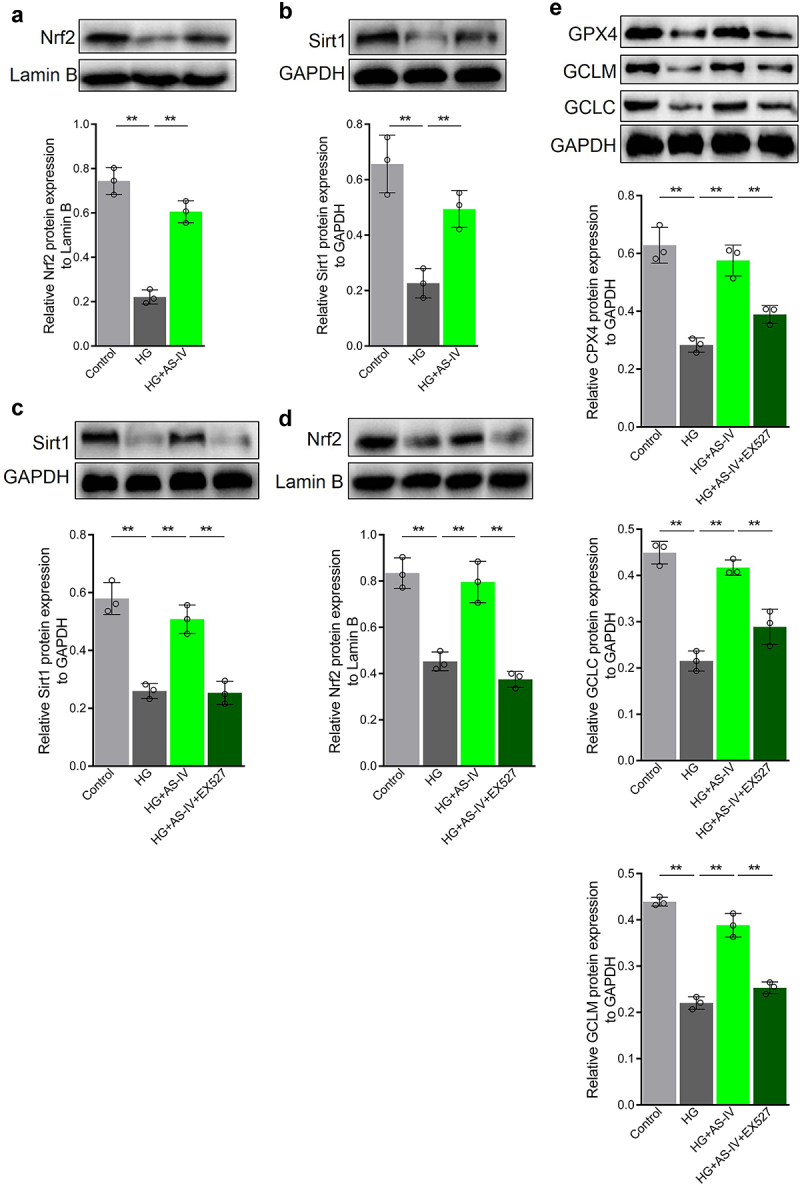
Astragaloside-IV (AS-IV) alleviates the damage caused by high glucose by reducing Sirt1/Nrf2 signaling activity in retinal pigment endothelial (RPE) cells. (a, d) Detection of Nrf2 by Western blot analysis. (b, c) Detection of Sirt1 by Western blot analysis. (e) Levels of glutathione peroxidase 4 (GPX4), glutamate cysteine ligase catalytic subunit (GCLC), and glutamate cysteine ligase modifier subunit (GCLM) proteins detected by Western blot analysis. ** indicates *p* values < 0.01.

High glucose conditions reduced Nrf2 protein levels in the nucleus of ARPE-19 cells. However, after the addition of AS-IV, Nrf2 level in the nucleus increased (Figure 4(a)). Sirt1 can positively regulate protein level and activity of Nrf2. High glucose decreased protein level of Sirt1 in ARPE-19 cells, whereas AS-IV could increase Sirt1 level (Figure 4(b)). On this basis, when Ex 527, a specific Sirt1 inhibitor was added to the cells, protein levels of Sirt1 in cells and Nrf2 in the nucleus decreased (Figure 4(c,d)). Further expression analysis of downstream Nrf2 target genes showed that protein expression levels of GPX4, GCLM and GCLC were decreased in ARPE-9 cells under high glucose conditions, and AS-IV could restore expression levels of these proteins, whereas Ex 527 could effectively inhibit the effect of AS-IV (Figure 4(e)). These results therefore suggest that Sirt1/Nrf2 signaling activity is reduced in RPE cells exposed to high glucose, thus resulting in decreased expression of antioxidant response molecules in RPE cells. Conversely, AS-IV can increase expression of antioxidant response molecules in RPE cells by enhancing Sirt1/Nrf2 signaling activity.

### HG inhibits Sirt1/Nrf2 signaling activity by increasing miR-138-5p level in RPE cells

**Figure 5.**
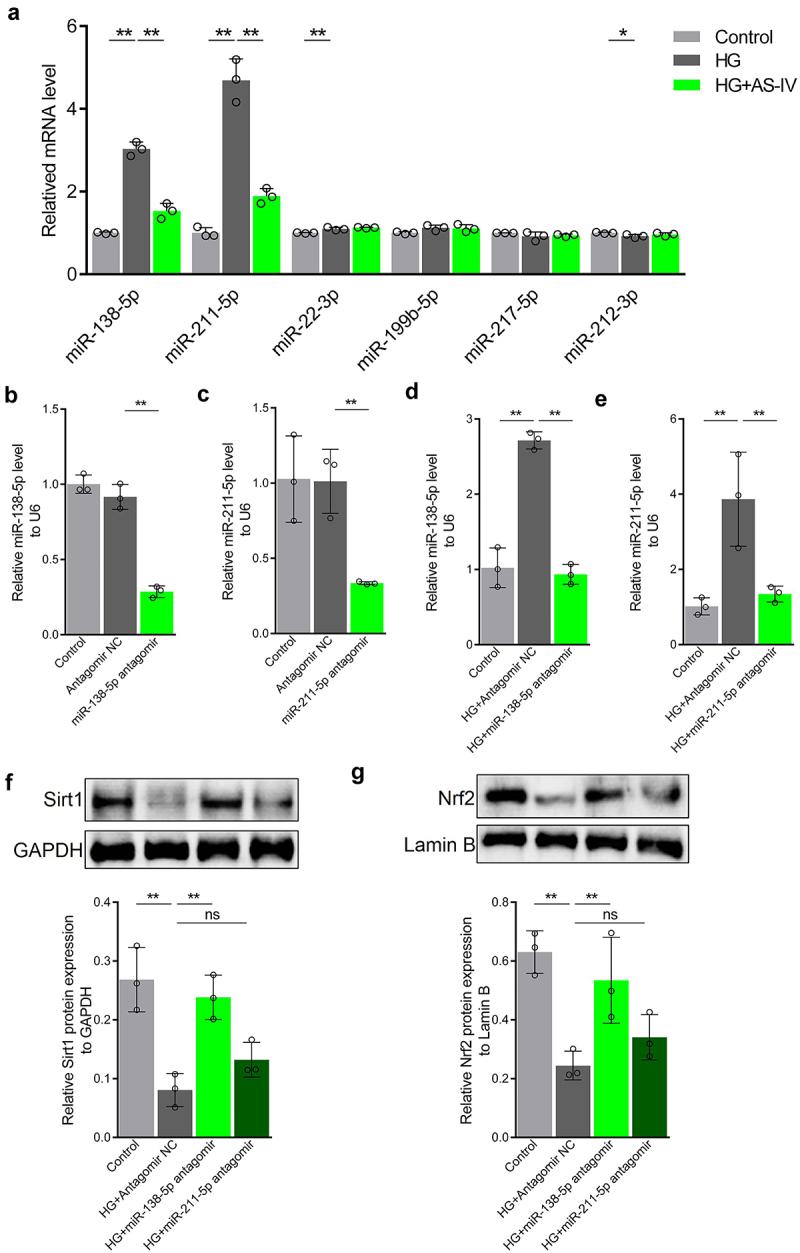
High glucose inhibits the Sirt1/Nrf2 signaling cascade activity by increasing expression of miR-138-5p in retinal pigment endothelial (RPE) cells. (a) Expression of miRNAs by quantitative reverse-transcription PCR (qRT-PCR). (b, d) Expression levels of miR-138-5p in RPE cells determined by qRT-PCR. (c, e) Expression levels of miR-211-5p in RPE cells detected by qRT-PCR. (f) Protein levels of Sirt1 detected by Western blot analysis. (g) Protein levels of Nrf2 in RPE cells detected by Western blot analysis. * indicates *p* values < 0.05; ** indicates *p* values < 0.01.

In order to elucidate whether miRNAs may regulate Sirt1 expression in RPE cells exposed to high glucose conditions or AS-IV, miRNA target prediction for Sirt1 was carried out using Targetscan and miRDB databases. Six miRNAs were selected basing on the following factors: (1) both Targetscan and miRDB could predict the interaction; (2) increased expression has been reported previously; (3) expression level decreased upon treatment with AS-IV; (4) oxidative stress response promoting effect. The selected miRNAs were miR-22-3p, miR-138-5p, miR-199b-5p miR-211-5p, miR-212-3p, and miR-217-5p. High glucose conditions increased the levels of miR-22-3p, miR-138-5p, and miR-211-5p in ARPE-19 cells, and decreased miR-212-3p level. After treatment with AS-IV, levels of miR-138-5p and miR-211-5p were restored to a certain extent (Figure 5(a)). In order to determine whether miR-138-5p and miR-211-5p mediate regulation of Sirt1/Nrf2 activity in RPE cells under high glucose conditions, firstly antagomirs of miR-138-5p and miR-211-5p were synthesized. After transfection into ARPE-19 cells, both inhibited the levels of miR-138-5p and miR-211-5p, respectively (Figure 5(b,c)). Furthermore, in cells under high glucose conditions, Sirt1 and Nrf2 levels in the nucleus were increased upon transfection of miR-138-5p antagomir, whereas transfection of miR-211-5p antagomir did not affect Sirt1 and Nrf2 levels (Figure 5(d–g)). These results indicated that high glucose can inhibit Sirt1/Nrf2 activity by increasing the level of miR-138-5p in RPE cells.

### AS-IV enhances Sirt1/Nrf2 signaling activity in RPE cells under high glucose conditions by inhibiting expression of miR-138-5p

**Figure 6.**
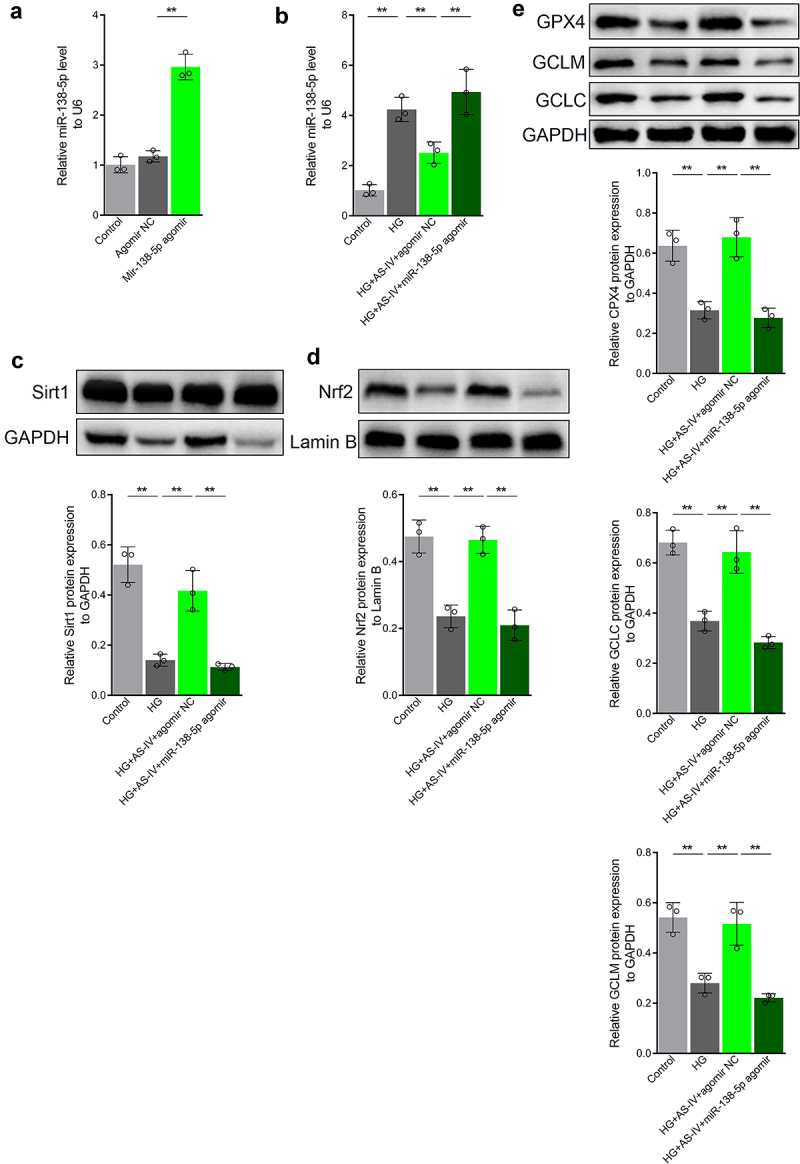
Astragaloside-IV (AS-IV) enhances Sirt1/Nrf2 signaling activity in retinal pigment endothelial (RPE) cells under high glucose conditions by inhibiting expression of miR-138-5p. (a, b) Expression levels of miR-138-5p in RPE cells detected by qRT-PCR. (c) Protein level of Sirt1 in RPE cells detected by Western blot analysis. (d) Protein level of Nrf2 in RPE nucleus detected by Western blot analysis and grayscale map. (e) Protein levels of glutathione peroxidase 4 (GPX4), glutamate cysteine ligase modifier subunit (GCLM) and glutamate cysteine ligase catalytic subunit (GCLC) in RPE cells detected by Western blot analysis. ** indicates *p* values < 0.01.

Since AS-IV could inhibit expression of miR-138-5p in RPE cells under high glucose conditions, it was speculated that AS-IV could enhance Sirt1/Nrf2 signaling activity in RPE cells under high glucose by inhibiting expression of miR-138-5p. To confirm this, the miR-138-5p antagomir was synthesized and then transfected to ARPE-19 cells. The level of miR-138-5p increased in transfected cells (Figure 6(a)). Furthermore, transfection of mir-138-5p antagomir decreased Sirt1 and Nrf2 levels (Figure 6(b–d)) as well as GPX4, GCLM and GCLC protein levels (Figure 6(e)). Thus, these results indicated that AS-IV can enhance Sirt1/Nrf2 signaling activity and promote expression of downstream antioxidant response molecules in RPE cells by inhibiting expression of miR-138-5p.

### AS-IV can decrease the rate of ferroptosis in RPE cells under high glucose by inhibiting expression of miR-138-5p

**Figure 7.**
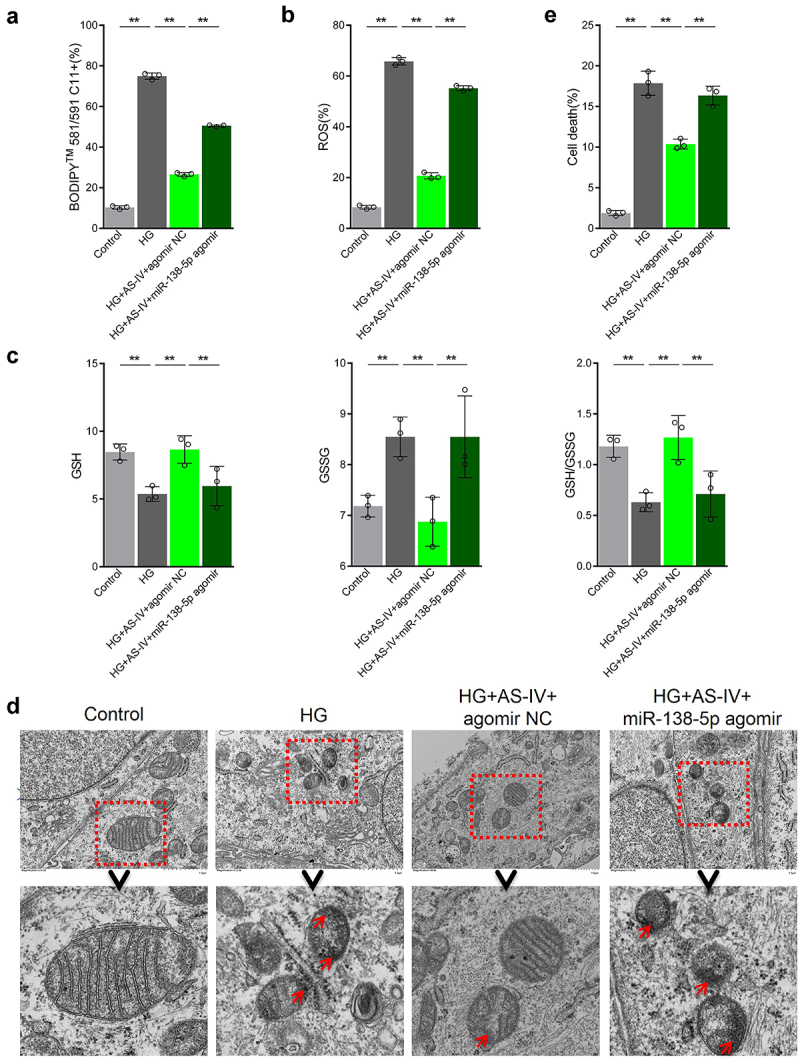
Astragaloside-IV (AS-IV) decreases ferroptosis in retinal pigment endothelial (RPE) cells under high glucose conditions by inhibiting expression of miR-138-5p. (a) Lipid oxidation level in cells detected by the C11-BODIPY™ 581/591 probe. (b) Total content of reactive oxygen species (ROS) detected by the CM-H2DCFDA probe. (c) Glutathione (GSH), oxidized glutathione (GSSG), and GSH:GSSG ratio analyses. (d) Cell substructure as determined by transmission electron microscopy (TEM); scale bar: 1.0 μm. (e) Cell death rate measured by flow cytometry. ** indicates *p* values < 0.01.

The effect of miR-138-5p on AS-IV in regulating the rate of ferroptosis in RPE cells under high glucose conditions was then investigated. The results showed that high glucose conditions increased lipid peroxidation as well as ROS and GSSG levels in RPE cells. In addition, high glucose decreased GSH content and the GSSG:GSH ratio, as well as reduced mitochondria size, decreased or abolished mitochondrial ridge, disrupted outer membrane, increased membrane density in certain areas, and increased cell death rate (Figure 7(a–e)). AS-IV treatment inhibited the negative effects of high glucose in RPE cells (Figure 7(a–e)), which was consistent with previous results. Moreover, transfection of miR-138-5p antagomir enhanced the negative effects induced by high glucose (Figure 7(a–e)). Collectively, these results indicate that AS-IV can reduce the rate of ferroptosis in RPE cells under high glucose conditions by inhibiting expression of miR-138-5p.

## Discussion

Oxidative stress leading to death of RPE cells is one of the main changes related to the pathological process of DR. Thus, basic medical research should focus on revealing the key mechanism underlying this phenomenon and finding suitable drugs to prevent oxidation-mediated RPE cell death. In the present study, high glucose was shown to induce ferroptosis in ARPE-19, which was related to the increase in miR-138-5p level and decrease in Sirt1/Nrf2 signaling activity. Conversely, AS-IV was shown to effectively inhibit miR-138-5p expression, increase Sirt1 and Nrf2 levels in the nucleus, and reduce the rate of ferroptosis thereby inhibiting death of RPE cells, which contributed to the understanding of the mechanisms underlying damage to RPE cells and the role of AS-IV in the prevention and treatment of DR.

Ferroptosis has been described in recent years as a newly discovered programmed cell death mode, being involved in cancer drug resistance, neurodegenerative diseases, ischemia/reperfusion, and other pathological processes [33–35]. Ferroptosis is therefore considered a potential target for disease treatment. Several ferroptosis inhibitors and inducers have been described, such as FIN56, sorafenib, SRS16-86, trolox and vitamin E. At present, studies have shown that ferroptosis occurs in RPE cells in age-related macular degeneration, and inhibition of ferroptosis is more effective in alleviating cell death than inhibition of apoptosis and necrosis pathways [8,9,36]. Although studies have shown that high glucose can induce ferroptosis in retinal microvascular endothelial cells [37], herein ferroptosis activity in RPE cells under high glucose conditions was described for the first time; ferroptosis activity was increased, therefore resulted in accumulation of ROS and decrease in GSH level, which eventually led to lipid peroxidation. Moreover, treatment with Fer-1 could inhibit cell death to a certain extent, indicating that ferroptosis could serve as an alternative therapeutic target for DR.

AS-IV, a high-purity natural product extracted from *A. membranaceus*, has remarkable antioxidant activity. In previous studies with RPE cells, AS-IV was shown to inhibit expression of miR-128 and the TRAF5 signaling pathway, thereby reducing the level of apoptosis induced by high glucose and isoflurane [16,38]. In adriamycin-induced cardiomyopathy, AS-IV inhibited ferroptosis, thereby inhibiting myocardial fibrosis and cardiac dysfunction [39]. In the present study, AS-IV was found to reduce lipid peroxidation level and ROS content in RPE cells under high glucose conditions, accompanied by decrease or absence of mitochondria, disruption of outer mitochondrial membrane, and increase in membrane density in some areas. Overall, AS-IV could alleviate the stimulating effect of high glucose on ferroptosis in RPE cells, which may contribute to the understanding of the possible role of AS-IV in DR treatment.

Nrf2 can bind to ARE in the promoter region of a variety of genes coding for antioxidant enzymes and promote expression of target genes, including *GPX, HO-1, GCLC* and *GCLM*, among others, while inhibiting ferroptosis in a variety of disorders by enhancing cellular antioxidant capacity [17,40,41]. Sirt1 is a class III protein deacetylase which promotes deacetylation of histone lysine residues and regulates the activity of many transcription factors, such as PGC-1α, NF-kB, P53, among others. Recent studies have shown that Sirt1 can promote expression and activity of Nrf2 through a variety of mechanisms, including deacetylation of PGC-1α and P53 as well as upregulation of transcription and activity of Nrf2 [24^,42–44^]. In the present study, high glucose reduced the expression levels of Sirt1 and Nrf2 in the nucleus of RPE cells, and inhibited expression of target genes *GPX4, GCLC* and *GCLM*, which are Nrf2 downstream genes. In contrast, AS-IV was shown to inhibit this phenomenon, and the addition of the Sirt1 inhibitor Ex 527 antagonized the effect of AS-IV, thus indicating that high glucose reduces protein level of Sirt1 and downregulates expression and activity of Nrf2. This may constitute an important mechanism for the decreased antioxidant capacity of RPE cells in DR. Therefore, AS-IV can play a protective role in cells by antagonizing the damaging process initiated by high glucose conditions.

At present, an increasing number of studies have suggested that AS-IV positively regulates Sirt1 at the protein and mRNA levels [45,46], including under high glucose conditions [26]. Moreover, Li Man and colleagues found that cycloastragenol, the active form of AS-IV, increased Sirt1 mRNA and protein levels rather than directly regulated its activity, in contrast to what was observed for resveratrol [45,47]. Therefore, the mechanism of AS-IV-mediated regulation of Sirt1 levels in RPE cells under high glucose conditions may partly be related to transcriptional activation and/or inhibition of Sirt1 mRNA degradation.

Diabetes has long-term impact on the occurrence and continuation of subsequent complications, a phenomenon called ‘metabolic memory’. Besides epigenetic modification, changes in miRNA level are one of the key contributors to the ‘metabolic memory’ mechanism. In RPE cells, the expression of Sirt1 is regulated by the abundance of its miRNA. Importantly, studies have found that AS-IV can increase expression level of Sirt1 by inhibiting the expression of miR-34a, therefore inhibiting the injury of cardiomyocytes under high glucose conditions [25]. These previous observations prompted us to explore the mechanism of AS-IV on the activation of Sirt1/Nrf2 signaling cascade in RPE cells under high glucose conditions from the perspective of miRNA expression.

miR-138-5p is known to regulate several pathological processes, such as myocardial injury, prostate cancer, intervertebral disc degeneration, among others, by targeting Sirt1 expression [^48–50^]. In the present study, it has been demonstrated for the first time that high glucose increased miR-138-5p level in RPE cells, inhibited expression of Sirt1, and reduced Nrf2 level in the nucleus. Collectively, these findings suggested that changes in miR-138-5p level might occur in RPE cells of DR patients, and could be considered as one of the key factors of ‘metabolic memory’ related to this disorder. In addition, AS-IV was found to inhibit miR-138-5p level in RPE cells under high glucose conditions and enhance Sirt1/Nrf2 activity. Thus, the current in-depth study described that AS-IV decreased ferroptosis in RPE cells exposed to high glucose conditions by downregulating miR-138-5p expression. Although it is not possible to exclude the possibility that AS-IV may affect Sirt1 expression in RPE cells in high glucose conditions at the transcriptional level, the findings presented herein partly indicated that AS-IV may delay disease progression by regulating miR-138-5p expression, possibly due to inhibition of Sirt1 degradation.

Therefore, it has been described herein that the expression of miR-138-5p increased in RPE cells under high glucose treatment, which led to reduced Sirt1/Nrf2 activity, reduced expression of antioxidant response-related molecules, increased ferroptosis, and promoted cell death. AS-IV was able to restore Sirt1/Nrf2 activity and expression of antioxidant-related molecules by inhibiting expression of miR-138-5p, therefore reducing ferroptosis and inhibiting high glucose-induced RPE cell death.

## Conclusion

Herein, high glucose was shown to increase ferroptosis in RPE cells. In addition, AS-IV could inhibit miR-138-5p expression, increase Sirt1/Nrf2 activity, and enhance cellular antioxidant capacity by antagonizing ferroptosis-related processes, thus reducing death of RPE cells. The results of the present study contribute to the understanding of the mechanism underlying high glucose-induced cell apoptosis, and indicate that AS-IV holds potential therapeutic value for DR.

## Data Availability

All data generated or analyzed during this study are included in this published article and its additional files.
